# The common stress responsive transcription factor ATF3 binds genomic sites enriched with p300 and H3K27ac for transcriptional regulation

**DOI:** 10.1186/s12864-016-2664-8

**Published:** 2016-05-04

**Authors:** Jonathan Zhao, Xingyao Li, Mingxiong Guo, Jindan Yu, Chunhong Yan

**Affiliations:** Department of Medicine, Division of Hematology and Oncology, Northwestern University Feinberg School of Medicine, Chicago, IL USA; Georgia Cancer Center, Augusta University, Augusta, GA USA; Department of Biochemistry and Molecular Biology, Medical College of Georgia, Augusta University, Augusta, GA USA; Center for Cell Biology and Cancer Research, Albany Medical College, Albany, NY USA

**Keywords:** ATF3, ChIP-seq, Enhancer, p300, H3K27ac, p53

## Abstract

**Background:**

Dysregulation of the common stress responsive transcription factor ATF3 has been causally linked to many important human diseases such as cancer, atherosclerosis, infections, and hypospadias. Although it is believed that the ATF3 transcription activity is central to its cellular functions, how ATF3 regulates gene expression remains largely unknown. Here, we employed ATF3 wild-type and knockout isogenic cell lines to carry out the first comprehensive analysis of global ATF3-binding profiles in the human genome under basal and stressed (DNA damage) conditions.

**Results:**

Although expressed at a low basal level, ATF3 was found to bind a large number of genomic sites that are often associated with genes involved in cellular stress responses. Interestingly, ATF3 appears to bind a large portion of genomic sites distal to transcription start sites and enriched with p300 and H3K27ac. Global gene expression profiling analysis indicates that genes proximal to these genomic sites were often regulated by ATF3. While DNA damage elicited by camptothecin dramatically altered the ATF3 binding profile, most of the genes regulated by ATF3 upon DNA damage were pre-bound by ATF3 before the stress. Moreover, we demonstrated that ATF3 was co-localized with the major stress responder p53 at genomic sites, thereby collaborating with p53 to regulate p53 target gene expression upon DNA damage.

**Conclusions:**

These results suggest that ATF3 likely bookmarks genomic sites and interacts with other transcription regulators to control gene expression.

**Electronic supplementary material:**

The online version of this article (doi:10.1186/s12864-016-2664-8) contains supplementary material, which is available to authorized users.

## Background

The development of human diseases is often accompanied by changes in the gene expression landscape. Regulated mainly at the transcription level, gene expression is tightly controlled by transcription factors (TF) that bind not only promoters proximal to transcription start sites (TSS), but also distal *cis*-regulatory elements (i.e., enhancers) that are far removed from TSS [1, 2]. Genome-wide profiling studies using chromatin immunoprecipitation coupled with sequencing (ChIP-seq) have identified thousands of functional/active enhancers that are either bound by the transcriptional co-activator p300, or characterized by their association with high levels of H3 K27 acetylation (H3K27ac) [3–5]. These enhancers often carry binding sites for more than one TF, which interact with the basal transcription machinery associated with core promoters to regulate gene transcription [2]. Very often, TFs also recruit chromatin-modifying enzymes to convert the chromatin to a state permissive for transcription. Pioneer transcription factors (e.g., FoxA1, PU.1), for example, are often the first to engage in a regulatory chromatin region upon stimulation, and enhance transcription by remodeling the local chromatin to make it competent for other TFs to bind [6]. While global profiling of genomic sites competent for TF binding is imperative for the understanding of TF functions, such work has also become increasingly important for defining disease etiologies, as mutations in *cis-*regulatory elements are frequently found to be associated with human diseases (e.g., cancer) by whole-genome sequencing studies [7].

Activating transcription factor 3 (ATF3) is a member of the ATF/CREB family of transcription factors involving in many important human diseases including cancer [8–11], atherosclerosis [12], infections [13], cardiac hypertrophy [14], and hypospadias [15]. The contributions of ATF3 to these diseases are often owing to its rapid induction by a wide-range of cellular stresses (e.g., DNA damage, oxidative stress, and endoplasmic reticulum (ER) stress), leading to activation of cellular signaling required for the maintenance of cell homeostasis. Indeed, while it binds and activates the tumor suppressor p53 in response to oncogenic challenges (e.g., DNA damage and *Pten* inactivation) [11, 16], ATF3 also engages in the immune response by interacting with NF-κB and repressing expression of proinflammatory cytokines induced by the toll-like receptor 4 [17]. Similarly, ATF3 induced by reactive oxygen species causes high susceptibility to secondary infections by repressing interleukin 6 (IL-6) expression during sepsis-associated immunosuppression [13]. Like other ATF/CREB transcription factors, ATF3 regulates transcription by binding the canonical ATF/CRE *cis*-regulatory element (5’-TGACGTCA-3’) or the similar AP-1 site (5’-TGA(C/G)TCA-3’) via its basic region-leucine zipper domain (bZip) [18]. Although an over-simplified model suggests that ATF3 homodimers and heterodimers (with other bZip proteins) repress and induce gene expression, respectively [19], the mechanism by which ATF3 regulates transcription remains largely unknown. Interestingly, although the structures of the bZip domains are highly similar allowing the largely diversified ATF/CREB proteins to bind the same *cis*-regulatory elements, the genes regulated by ATF3 are distinct from those controlled by its family members. ATF3 and ATF6, for instance, regulate expression of proapoptotic genes and genes involved in protein folding and ER quality control upon ER stress, respectively [20]. As recent evidence supports that ATF3 engages in a complex protein-protein interaction network involving many TFs and transcription co-regulators [16, 21, 22], it is likely that the interactions with other nuclear proteins define the genomic sites where ATF3 binds and the transcription programs that ATF3 regulates. Characterization of genome-wide ATF3 binding sites would thus lead to further elucidation of the ATF3 interaction network and a better understanding of how ATF3 regulates expression of disease-associated genes.

In this study, we present the first comprehensive analysis of ATF3 binding profiles in the human genome. We show that ATF3 bound a large portion of active enhancers characterized by p300 binding and enriched with K27 acetylated histone H3 (H3K27ac) under the basal condition where ATF3 was expressed at a very low level. While the expression of genes proximal to these enhancers tended to be regulated by ATF3, ATF3 was co-localized with p53 and regulated p53-target gene expression in response to DNA damage. Our results thus suggest that ATF3 likely bookmarks genes for transcriptional regulation under basal and stressed conditions.

## Results

### Genome-wide mapping of ATF3 binding sites using isogenic cell lines

**Fig. 1:**
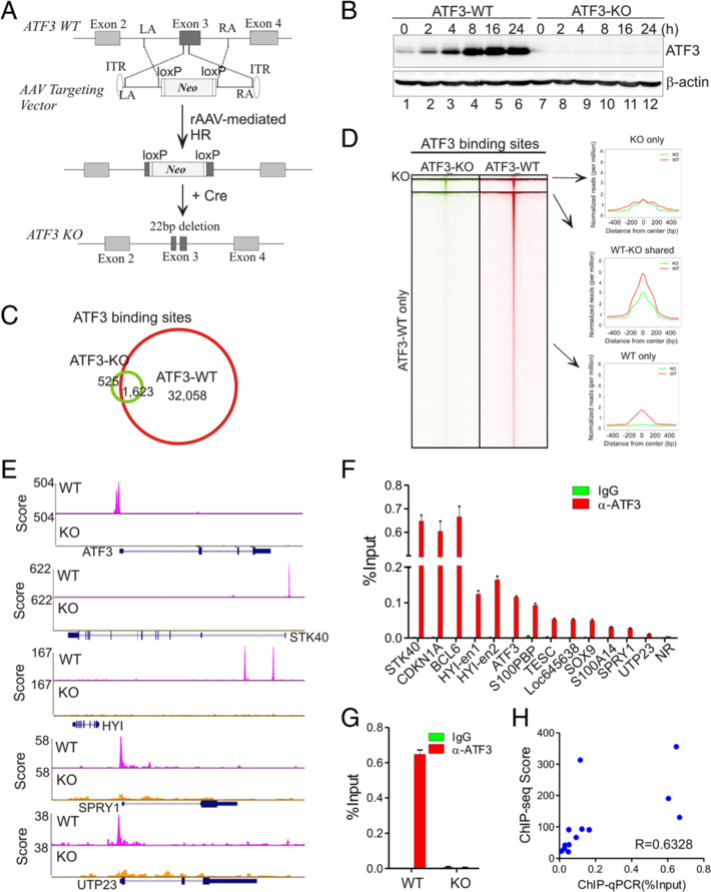
ATF3 binding profiling using isogenic HCT116 cells. **a** rAAV-mediated genome editing was applied to generate ATF3-knocked out HCT116 cells. rAAV-mediated homologous recombination led to insertion of the AAV targeting vector into *ATF3* exon 3. A deletion of 22 bp was generated in one *ATF3* allele after Cre-mediated excision of the *Neo* selection gene. LA and RA, left and right homology arms; ITR, inverted terminal repeat; KO, knockout. **b** ATF3 expression was completely abolished in ATF3-KO cells. Indicated cells were treated with 1.5 μM of CPT and subjected to Western blotting. **c** Venn diagram showing ATF3-binding peaks in ATF3 wild-type (ATF3-WT) and knockout (ATF3-KO) cells. **d** Heatmap and intensity plots showing ATF3 peaks in ATF3 WT and KO cells. **e** Representative genome browser views of ATF3 peaks. ATF3 peaks near *ATF3*, *STK40*, *HYI*, *SPRY1*, and *UTP23* were shown for both ATF3-WT and KO cells. **f, g** ChIP-qPCR was used to validate ATF3 binding to representative genome sites that were referred to as the names of their annotated genes. NR, no-binding control region. Error bars represent SD for three replicate measurements. **h** The binding intensity determined by independent ChIP-qPCR assays was correlated with ChIP-seq scores of peaks tested in (**f**) and (**g**)

To profile global ATF3-binding sites, we first employed a genome-editing approach based on recombinant adenoassociated viruses (rAAV) to generate a cell line in which *ATF3* expression was knocked out. Towards this end, we constructed an AAV targeting vector containing left (LA) and right homology arms (RA) flanking the exon 3 of the *ATF3* gene, and introduced the vector into HCT116 human colon cancer cells via rAAV infections [23]. Homologous recombination between the homology arms and the *ATF3* fragments resulted in the insertion of a selection gene (TK-neo) into an *ATF3* allele. A small deletion (22 bp) in the exon 3 was subsequently generated by Cre-mediated excision of the selection gene (Fig. 1a). The same strategy was employed to target the second ATF3 allele, generating a cell line (ATF3-KO) in which *ATF3* expression was disrupted. We confirmed that ATF3 was not expressed and ATF3 expression was not induced by camptothecin (CPT) - a DNA-damaging agent - in the knockout cells (Fig. 1b).

We thus subjected the wild-type (ATF3-WT) cells and the knockout cells to chromatin immunoprecipitation using an ATF3 antibody. Precipitated DNAs were then labeled and subjected to next-generation sequencing and sequencing reads were mapped to human genome and analyzed for enrichment. Although ATF3 was expressed at a low level (Fig. 1b, lane 1), we identified 33,681 high-confident ATF3-binding peaks in the sample derived from ATF3-WT cells (Fig. 1c). Out of them, a majority of peaks (32,058) were ATF3 specific, as they were not found in the ATF3-KO cells (Fig. 1c and d). A few examples of ATF3 peaks were shown in Fig. 1e. Of note, like a majority of identified sites, these ATF3 peaks were found only in the ATF3-WT sample but not in the ATF3-KO sample (Fig. 1e). Consistent with an early result that ATF3 represses its own expression [24], we found that ATF3 strongly bound its own promoter (Fig. 1e). Using quantitative PCR (qPCR) to examine samples from an independent ChIP experiment, we confirmed that ATF3 bound to all of the tested genomic sites identified by ChIP-seq (Fig. 1f). Again, ATF3 bound to its own promoter in the ATF3-WT cells but not in the knockout cells (Fig. 1g). The strengths of ATF3 binding to these sites measured by ChIP-qPCR were well correlated with the ChIP-seq scores (R = 0.6328), demonstrating high reproducibility and reliability of our ChIP-seq data.

### Global ATF3-binding profile and motif analysis

**Fig. 2:**
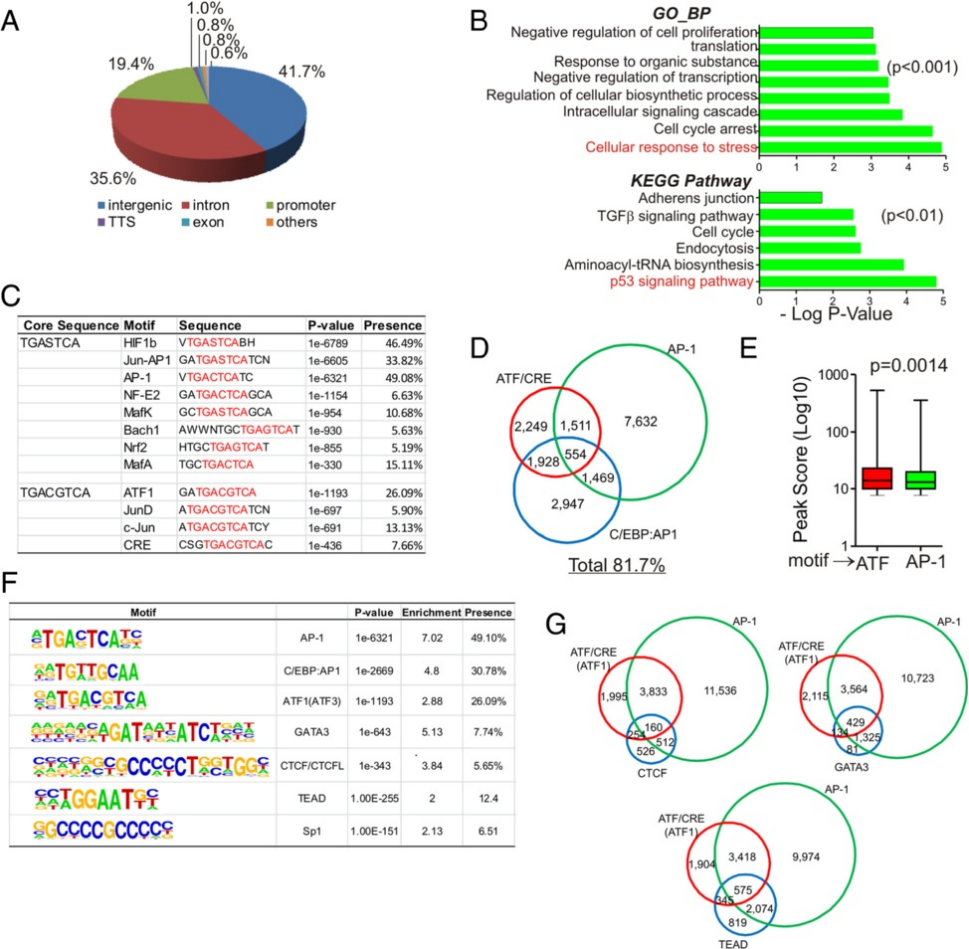
Global ATF3-binding profile under the basal condition. **a** A pie chart showing distribution of ATF3 binding sites relative to annotated genes. **b** 600 annotated genes with top peak scores were used for DAVID GO analysis. GO biological process (BP) terms and KEGG pathway terms are shown. **c** Top motifs identified in the ATF3 binding sites. **d** Schematic showing relative abundance and overlaps of the three known ATF3 binding motifs. The numbers are peak numbers. **e** The binding of ATF3 to the ATF/CRE motif appeared to be stronger than the AP-1 motif. Student *t*-test. **f** A table showing top motifs after combining motifs with same/similar sequences. **g** Venn diagram showing overlaps of CTCF, GATA3, and TEAD motifs with the ATF3 motif

The 32,058 ATF3-specific peaks were annotated to 10,262 unique genes. We analyzed the distribution of these binding sites relative to TSS in the human genome. Consistent with the ATF3’s role as a transcription factor, about one fifth (19.4 %) of the ATF3 peaks were localized in promoters, which was defined as regions that were ±2 kb surrounding TSS (Fig. 2a). Given that only a small portion of DNA in the whole genome can be defined as promoters, these results indicate that ATF3 were enriched in promoters. However, ATF3 also bound genomic regions far removed from TSS (Fig. 2a), suggesting that ATF3 also likely regulates transcription via long-range interactions. Gene Ontology (GO) analysis of the top 600 annotated genes with high binding scores revealed that ATF3 preferably bound to regulatory elements for genes involving in biological processes such as cellular response to stress, cell cycle arrest and intracellular signaling cascade, as well as pathways such as p53 signaling pathway (Fig. 2b). Interestingly, “cellular response to stress” and “p53 signaling pathway” turned out to be the top GO terms for the ATF3-bound genes, consistent with the well-established roles that ATF3 plays in regulating cellular stress responses and the p53 pathway [16, 18].

We also searched the ATF3 binding sites for known TF binding motifs using the Homer *de novo* motif discovery software. A total of 140 motifs were identified with a p value smaller than 0.01. With only one exception (CEBP:AP1 motif), the top 12 identified motifs contained either the canonical ATF/CRE sequence (i.e., 5’-TGACGTCA-3’) or the AP-1 sequence (i.e., 5’-TGASTCA-3’, S = C/G) (Fig. 2c). As C/EBP harbors a bZip domain that can mediate dimerization with other bZip proteins including ATF3 [25], it might be that ATF3 bound the CEBP:AP1 motif through dimerization with C/EBP. Overall, 81.7 % of ATF3 binding sites contain an element predicted to be bound by ATF3 - collectively referred to as the ATF3 motif hereafter (Fig. 2d) - suggesting that ATF3 directly binds genomic DNA in most cases. Interestingly, although more ATF3 peaks contained the AP-1 motif (Fig. 2e), the binding affinity of ATF3 to the canonical ATF/CRE element appeared to be higher than that for ATF3 binding to the AP-1 element (Fig. 2e). In addition to these known ATF3 binding motifs, other top ATF3 binding motifs (*i.e.*, Enrichment > 2) include GATA3, CTCF, TEAD, and Sp1, which was presented in 7.7 %, 5.6 %, 14.8 %, and 6.5 % of ATF3 peaks, respectively (Fig. 2f). Although these ATF3-binding peaks often contain a known ATF3 motif (Fig. 2g), ATF3 might also bind these motifs through interacting with corresponding TFs. Indeed, ATF3 has been shown to interact with Sp1 [26].

### ATF3 globally binds active enhancers enriched with p300 and H3K27ac

**Fig. 3:**
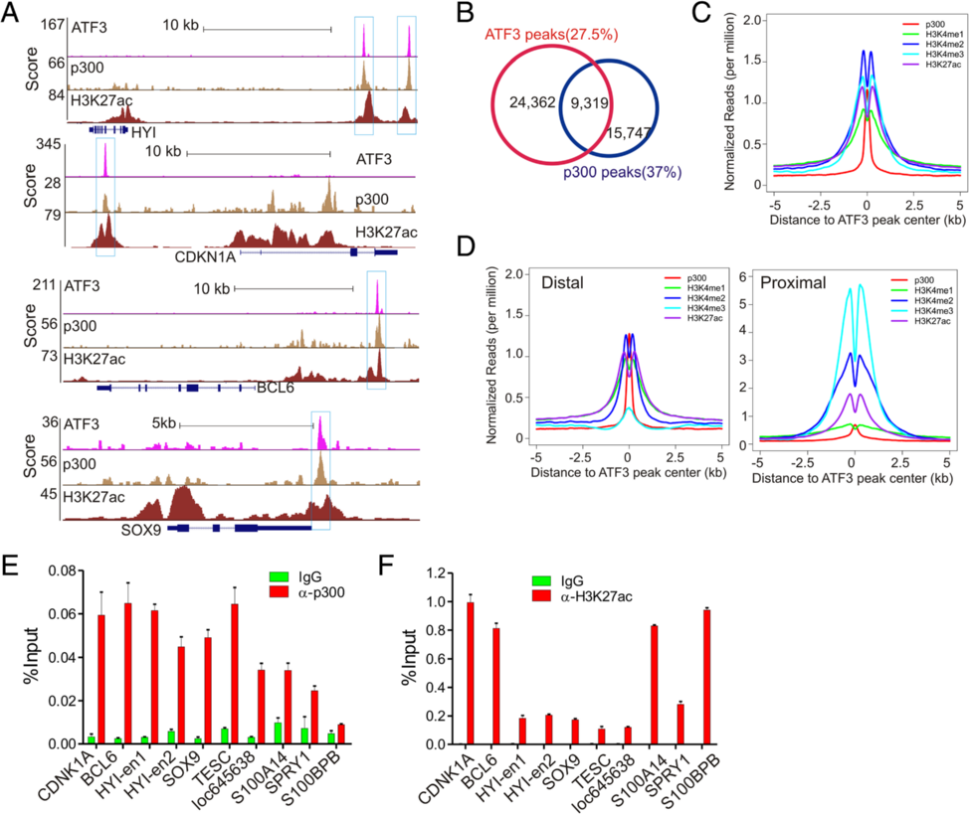
ATF3 globally binds genomic sites enriched with p300 and H3K27ac. **a** Genome browser views showing co-localization of ATF3 with p300 and H3K27ac in several representative genomic sites. **b** Venn diagram showing overlaps between ATF3 peaks and p300 peaks. **c** Intensity plot showing co-localization of ATF3 with p300 and H3K27ac. **d** Venn diagram showing overlaps between p300 distal peaks (active enhancers) and ATF3 peaks. **e** ChIP-qPCR validation of p300 binding to the ATF3 binding sites. HCT116 cells were subjected to ChIP using a p300 antibody. Precipitated DNA was quantitated using qPCR. **f** H3K27ac was enriched in the ATF3-binding sites. ChIP-qPCR was carried out to determine H3K27ac levels in the ATF3-binding sites

As ATF3 bound genomic sites far removed from TSS, we sought to determine whether these sites are coincided with active enhancers that are often marked by p300 binding and flanked with high levels of H3K27ac [3–5]. Towards this end, we acquired p300, H3K27ac, H3K4me1, and H3K4me3a ChIP-seq data (HCT116 cells) from the Gene Expression Omnibus (GEO) database GSE51176 and GSE38447 [27, 28]. We first examined the ATF3 peaks in the Genome Browser, and found that ATF3 bound to many sites that were also bound by p300 and flanked by regions with high levels of H3K27ac (Fig. 3a), suggesting that ATF3 bound to active enhancers. Indeed, unbiased statistics analysis revealed that up to 27.5 % of ATF3 peaks were overlapped with p300 peaks, and 37 % of p300 peaks were bound by ATF3 (Fig. 3b). Intensity plots also show that p300 was globally co-localized with ATF3 and that the H3K27ac histone marker surrounded the ATF3/p300 peaks as expected (Fig. 3c). We segregated the ATF3 peaks into proximal sites (within 2 kb) and distal sites (>2 kb) based on their distances to TSS, representing H3K4me3-enriched promoters and H3K4me1-enriched enhancers, respectively (Fig. 3d). The intensity plots revealed that it was the distal sites, but not the proximal sites, that were coincided with p300 binding events (Fig. 3d). Using qPCR, we validated that p300 bound to all of the tested ATF3 binding sites in an independent ChIP experiment (Fig. 3e). Similarly, the enrichment of H3K27ac in these ATF3 sites was also validated (Fig. 3f). Of note, as p300 is not the only enzyme that can acetylate H3 at the K27 site, the H3K27ac level was not strictly correlated with the p300 level in some genome sites. Taken together, our results have revealed that a large portion of ATF3 bound active enhancers.

### ATF3-regulated gene expression correlates with ATF3 enhancer binding

**Fig. 4:**
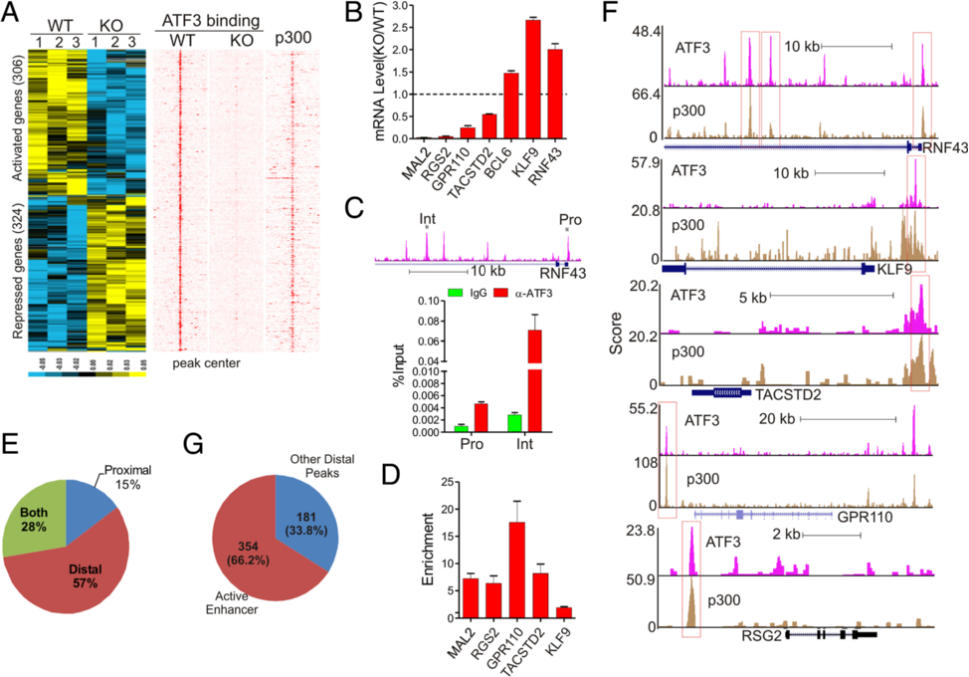
Binding of ATF3 to active enhancers correlates with ATF3-regulated gene expression. **a** Heatmaps showing ATF3-regulated genes, and their binding by ATF3 and p300. **b** qRT-PCR validation of genes differentially expressed between ATF3-wildtype and knockout cells identified by microarray. **c, d** Validation of ATF3 binding to differentially-expressed genes by ChIP-qPCR. **e** ATF3 was localized in regions distal to TSS (>2 kb) of differentially-expressed genes. **f** Representative genome browser views of co-localization of ATF3 and p300 in ATF3-regulatd genes. **g** ATF3 was localized in active enhancers of ATF3-regulated genes. Error bars represents SD

An interesting question surfaced as to how ATF3 binding to genomic sites regulates gene expression. To address this question, we subjected the ATF3-wildtype and knockout cells to cDNA microarray assays. Although ATF3 bound to 10,262 genes, only 1,087 unique genes, including several known ATF3 targets *(i.e.*, *ASNS*) [29], were differentially expressed between the WT and KO cells (FDR < 0.05, Additional file 1: Figure S1A and S1B). Among these genes, 630 (60 %) were bound by ATF3 and thus more likely to be directly regulated by ATF3 (Fig. 4a). Roughly equal numbers of genes was either activated or repressed by ATF3 (Fig. 4a), suggesting that ATF3 can function as both a transcription repressor and a transcription activator. In line with the reported tumor suppressor role in colon cancer [30, 31], ATF3 appeared to induce expression of genes involving in mitosis and stress responses while repressing genes regulating vasculature development, migration, and apoptosis (Additional file 1: Figure S1C). We validated 7 differentially-expressed genes by quantitative RT-PCR (Fig. 4b) and their binding by ATF3 by independent ChIP-qPCR assays (Fig. 4c and d). Interestingly, although ATF3 were often reported to regulate gene expression by binding to a ATF3 motif localized in promoters, only 15 % (95/630) of the ATF3-regulated genes identified herein were bound by ATF3 exclusively at their promoters (proximal genomic regions) (Fig. 4e). The rest of genes either were bound by ATF3 exclusively at distal regions (57 %, or 361/630), or at both promoters and distal regions (28 %, or 95/630) (Fig. 4e). These results suggest that ATF3 could regulate gene expression by binding to distal *cis*-regulatory elements localized in active enhancers. Indeed, except *MAL2*, all other validated ATF3-target genes were bound by ATF3 at distal regions overlapped with p300 peaks (Fig. 4f). Of the 535 genes containing distal ATF3-binding sites, 354 (66.2 %) were associated with active enhancers enriched with p300 and bound by ATF3 (Fig. 4g). Interestingly, ATF3-repressed genes appeared to be more likely to harbor distal ATF3 regulatory elements than ATF3-activated genes (Additional file 1: Figure S1D), although the TF motifs contained in the ATF3-binding sites in these two groups of genes were similar (Additional file 1: Figure S1E).

### DNA damage alters the ATF3-binding landscape for gene regulation

**Fig. 5:**
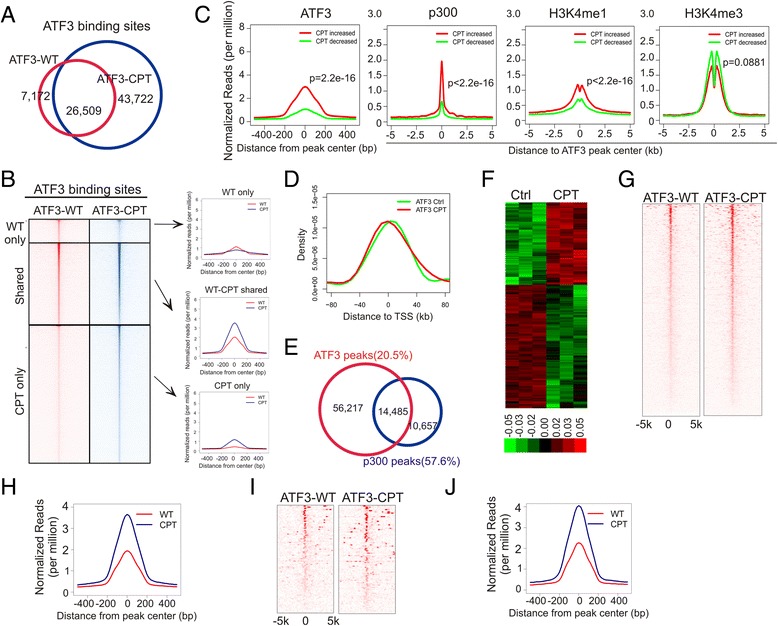
DNA damage alters the ATF3-binding landscape for transcriptional regulation. **a** Venn diagram showing overlap of ATF3-binding peaks between the basal (ATF3-WT) and the CPT-treated (ATF3-CPT) conditions. **b** Heatmaps and intensity plots showing alterations in the ATF3-binding profile caused by DNA damage. **c** Intensity plots showing different enrichments of ATF3, p300, and histone markers between CPT-induced and decreased peaks. **d** Distribution of ATF3-binding sites under basal (ATF3-Ctrl) and stressed (ATF3-CPT) conditions. **e** Venn diagram showing overlaps of ATF3 peaks and p300 peaks under the CPT-treatment condition. **f** Heatmaps showing CPT-regulated genes. **g** Heatmaps of ATF3-binding sites associated with CPT-regulated genes showing that ATF3 was pre-loaded on most of these genes before stress. **h** Intensity plot showing that ATF3 binding to CPT-regulated genes was increased by the CPT treatment. **i** Heatmap showing ATF3-regulated genes under the DNA damage condition were pre-bound by ATF3. **j** Intensity plot showing that DNA damage increased ATF3 binding to ATF3-regulated genes

As a common stress sensor, ATF3 may regulate cellular stress responses by altering the gene expression landscape. To understand how cellular stresses alter genome-wide ATF3 binding profile for transcriptional regulation, we subjected HCT116 cells treated with CPT for ChIP-seq assays. As CPT could increase the ATF3 expression level (Fig. 1b) [32], it was not surprising that the DNA-damaging treatment increased the number of ATF3-binding sites to 70,231 (Fig. 5a) – one fold more than that under the basal condition. However, we found that a large number of sites (7,172, 21.3 %) bound by ATF3 under the basal condition were not detected after the CPT treatment (Fig. 5a and b, “WT-only”). ATF3 bound these sites more weakly than the remained sites (Fig. 5b, “WT-only” vs. “Shared” peaks, *p* = 7.46e-05). Of the “shared” peaks, DNA damage increased ATF3 binding to 13,253 sites but decreased its binding to the rest 13,256 sites (Additional file 1: Figure S2A). Interestingly, while the CPT-increased sites appeared to be bound by ATF3 more strongly than the CPT-decreased sites under the basal condition (Fig. 5c), the increased sites were also often bound by p300, or enriched with H3K4me1, suggesting that DNA damage promoted ATF3 to bind to active enhancers (Fig. 5c). In contrast, CPT tended to decrease ATF3 binding to those sites localized in promoters and thus often flanked by a high level of H3K4me3 [33] (Fig. 5c). Consistent with these observations, although DNA damage did not significantly change the overall genome distribution and motif composition of the ATF3 binding sites (Additional file 1: Figure S2B and S2C), it promoted ATF3 to bind to the sites distal to TSS (Fig. 5d). Similarly, DNA damage increased the number of sites bound by both ATF3 and p300, and the number of these ATF3-bound active enhancers was increased from 37 % under the basal condition to 57.6 % upon stress (Fig. 5e). Interestingly, the new sites bound by ATF3 after DNA damage (“CPT only” in Fig. 5b) had weaker ATF3-binding affinities than the sites bound by ATF3 under the basal condition (*p* = 1.73e-07, comparing “CPT only” vs “Shared” peaks in Fig. 5b), but had stronger affinities than those lost peaks (*p* = 0.000345, “CPT only” vs “WT only”, Fig. 5b). Our results thus suggest that ATF3 not only increased its level, but also altered its genome binding in response to DNA damage.

We next addressed the question as to what changes in gene expression the altered ATF3-binding would cause under the DNA damage condition. Treating HCT116 cells with CPT for 4 h resulted in an increase in expression of 733 genes and a decrease in expression of 1095 genes (fold > 1.5, *p* < 0.05) (Fig. 5f). 1,300 (71.1 %) of these altered genes were bound by ATF3 after DNA damage (Fig. 5g), and thus were more likely regulated by ATF3. Interestingly, 82.9 % of ATF3-bound, CPT-regulated genes were also bound by ATF3 before the cells were treated with CPT (Fig. 5g), suggesting that ATF3 were pre-loaded on the genomic sites for gene regulation under stressed conditions. However, stressed ATF3 appeared to bind these sites more strongly than the quiescent protein (Fig. 5h). Given that CPT equally increased or decreased ATF3 binding on the “shared” sites (see above), these results indicate that DNA damage selectively promoted ATF3 to bind to genomic sites associated with regulated genes.

To further determine the relationship between ATF3 binding and gene regulation under the stressed condition, we analyzed the gene expression data for ATF3 knockout cells, and generated a curated list of 93 genes that were judged, with high confidence, as ATF3-regulated genes in response to DNA damage (Additional file 1: Figure S2D), based on (1) that fold changes before and after the CPT treatment were significantly different (*p* < 0.05, paired *t*-test) between ATF3-wildtype and -knockout cells, (2) that the genes bound by ATF3 with a small binding score (<10) and thus more likely to be derived from experimental errors were excluded. Once again, while 82 (88.2 %) of these genes had already been bound by ATF3 under the basal condition, CPT further increased ATF3 binding to these regulated genes, regardless whether their expression was induced or repressed by CPT (Fig. 5i and j). Interestingly, about a half (43, or 46 %) of these genes contained one or more active enhancers that were bound by both p300 and ATF3 (Fig. 5i), consistent with our previous conclusion that ATF3 can bind to active enhancers to regulate gene expression.

### ATF3 collaborates with p53 in regulating target gene expression

**Fig. 6:**
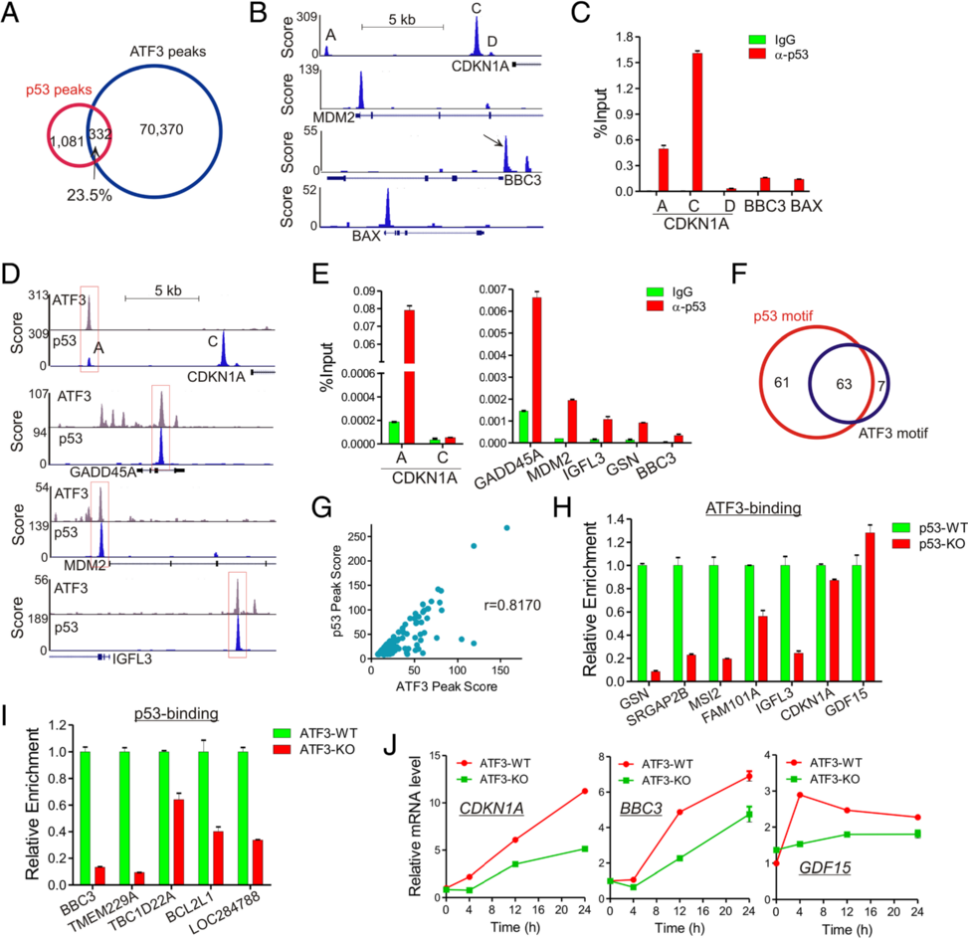
Co-localization of ATF3 and p53 in genomic sites regulates gene expression in the DNA damage response. **a** Venn diagram showing the overlap between ATF3 peaks and p53 peaks under the DNA damage condition. **b** Genome browser views of p53 binding to several well-characterized p53 target genes. **c** Binding of p53 to indicated sites was validated by independent ChIP-qPCR assays. **d** Genome browser views of co-localization of ATF3 and p53 in representative genomic sites. **e** ATF3 and p53 were co-localized in genomic sites as demonstrated by re-ChIP assays. HCT116 cells treated with 1.5 μM of CPT for 4 h were first subjected to ChIP using the ATF3 antibody. The chromatin precipitated by the ATF3 antibody was then eluted from agarose beads, and subjected to the second round of ChIP using the p53 antibody. qPCR assays were used to quantitate re-ChIPed DNA. **f** Venn diagram showing the overlap of p53-binding sites containing the p53 motif or the ATF3 motif. **g** The ATF3 peak score correlated with the p53 peak score in the sites co-localized by ATF3 and p53. **h** ATF3 binding was often decreased in p53-knockout cells. p53-wildtype and knockout (p53-KO) HCT116 cells were subjected to ChIP-qPCR to measure binding of ATF3 to the indicated sites. **i** p53 binding was decreased in ATF3-knockout cells. ATF3-wildtype and knockout (ATF3-KO) HCT116 cells were subjected to ChIP-qPCR to measure binding of p53 to the indicated sites. **j** Expression of p53 target genes was repressed in ATF3-KO cells. Indicated cells were treated with 1.5 μM of CPT for qRT-PCR assays. ATF3 binding to these genes before and after CPT treatments in ATF3-WT cells were shown in Additional file 1: Figure S3

p53 is a master transcription factor that transactivates genes (e.g., *CDKN1A* and *BBC3*, best known as *p21* and *PUMA*, respectively) essential for driving cellular responses (*e.g.*, cell cycle arrest and apoptosis) to DNA damage [34]. As ATF3 can bind p53 [16] and we also found that ATF3-bound genes engage in the p53 signaling pathway (Fig. 2c), we sought to determine how ATF3 interacts with p53 at genomic sites to regulate gene expression in response to CPT-induced DNA damage. We first profiled global p53 binding by subjecting CPT-treated HCT116 cells to ChIP-seq analysis. We identified 1,412 p53-binding peaks (Fig. 6a), a number which was low but within the same range (from 743 to 4,785) as other reports [35–37]. These identified binding sites included 3 previously-characterized p53-binding sites in the *CDKN1A* enhancer and promoter regions (site A, C and D, respectively) (Fig. 6b) [35], and sites localized in the promoters of well-characterized p53 target genes *MDM2*, *BBC3* and *BAX* (Fig. 6b). These p53-binding sites were validated by independent ChIP-qPCR assays (Fig. 6c). Consistent with the notion that ATF3 is a p53 regulator [16], we found that ATF3 bound to 23.5 % (332) of p53-binding sites (Fig. 6a), including the *CDKN1A* site A (but not site C and D), and the sites associated with *GADD45A*, *MDM2* and *IGFL3* (Fig. 6d). Using re-ChIP assays, we confirmed that ATF3 was co-localized with p53 at Site A, but not Site C, of *CDKN1A*, and other tested genomic sites associated with *GADD45A*, *MDM2*, *IGFL3*, *GSN*, and *BBC3* (Fig. 6e). Of these ATF3/p53 co-localized sites, 61 only carried a p53 motif, 7 only carried an ATF3 motif, and 63 harbored both motifs (Fig. 6e; Additional file 2: Table S1). Given that ATF3 can directly bind p53 [16], co-localization of ATF3 with p53 at genomic sites might be owing to p53-mediated recruitment of ATF3 to sites containing the p53 motif, and/or ATF3-mediated recruitment of p53 to sites harboring the ATF3 motif. Indeed, we found a strong correlation between the ATF3-binding score and the p53 peak score at these genomic sites (r = 0.8170, Fig. 6g). Moreover, p53 depletion dramatically impaired ATF3 binding to 5 out of 7 tested p53-motif-only sites (Fig. 6h). Of note, although p53 was previously shown to be required for ATF3 induction by DNA damage caused by γ-irradiation [38], we did not see decreased ATF3 expression in p53-knockout cells under our experimental condition (data not shown). The reason why p53 knockout did not decrease ATF3 binding to the *CDKN1A* Site A and the GDF15 p53-binding site that lacked the ATF3 motif was unclear, but other TFs might recruit ATF3 to these sites. Interestingly, p53 binding to the sites containing only the ATF3 motif was significantly decreased by ATF3 knockout as well (Fig. 6i), suggesting that ATF3 could also recruit p53 to genomic sites that do not contain a p53 motif. Thus, the ATF3-p53 interaction might expand the list of genes that can be regulated by p53. Interestingly, 19.5 % (58/297) of the ATF3/p53 co-localized sites, including the site associated with *CDKN1A*, *BBC3* and *GDF15*, were also enriched with p300, suggesting that many of these sites were active enhancers and thus the ATF3-p53 interaction on genomic sites were likely functional. Indeed, we demonstrated that knockout of ATF3 expression impaired CPT-induced *CDKN1A*, *BBC3,* and *GDF15* expression (Fig. 6j). Therefore, our results indicate that ATF3 could interact with p53 at genomic sites thereby regulating gene expression in the DNA damage response.

## Discussion

It is often shown that ATF3 binds the ATF/CRE *cis*-acting element localized in gene promoters and regulate expression of genes associated with human diseases [12–14]. We carried out this study in light of the fact that a genome-wide ATF3-binding profile in the human genome was lacking. Employing engineered ATF3-knockout cells as the specificity control, we identified 33,681 specific ATF3-binding sites across the human genome under the basal condition. Although this number was surprisingly large given that the basal ATF3 expression level was low, it was comparable to 22,521 sites identified in mouse dendritic cells [39]. As 81.7 % of the ATF3-binding sites contained a known ATF3 motif (Fig. 2e) [40], ATF3 might directly bind a majority of these sites. It was thus likely that the low level of constitutively-expressed ATF3 was sufficient to bind most of available sites in the genome. Interestingly, ATF4, a family member sharing the same binding motif with ATF3, binds only 1,210 sites in the mouse genome [39]. While this difference might be owing to different DNA-binding affinity, interactions with other transcription regulators could poise ATF3 for a higher level of genome binding. The latter possibility is supported by the fact that ATF3 differs from ATF4 in its ability of interacting with other proteins [25]. It is worth noting that the ATF3 genome-occupancy level is lower than that of pioneer factors, which often bind more than 50,000 genomic sites [39], but significantly higher than that of most of gene-specific TFs (*e.g.*, p53) that generally occupy a few thousands of genomic sites (Fig. 6a). It is thus tempting to hypothesize that ATF3 serves as a molecular beacon, or “primer factor”, that binds genomic sites subsequent to binding of pioneer factors, and directs other TFs or transcription co-regulators to appropriate genomic sites upon stimulation [39]. This hypothesis was partly supported by the findings that ATF3 directly interacts with many TFs (e.g., p53, p63, AR, Sp1) [16, 21, 26, 41] and histone modifying enzymes (e.g., Tip60 and HDAC) [17, 22]. Importantly, while the GO analysis revealed that the ATF3-bound genes were associated with cellular response to stress under the basal condition (Fig. 2c), we found that the genes whose expression was regulated by DNA damage were often pre-bound by ATF3 (Fig. 5g). Thus, like the transcription factor p63 [42], ATF3 might bookmark genes for transcriptional regulation. In this regard, it is likely that ATF3 recruits diverse sets of TFs to genomic sites pre-bound by ATF3 upon varying stimuli, thereby regulating gene expression and mounting rapid, appropriate responses to varying cellular stresses. However, DNA damage-induced changes in ATF3 binding were more dynamic than what the “primer-factor” hypothesis suggests [39]. DNA damage not only increased the number of ATF3 binding sites by 1 fold, but abolished up to one-fifth of the basal binding events (Fig. 5b). In addition, CPT increased ATF3 binding to some genomic sites but decreased its binding to almost equal numbers of other sites. While stress-induced loss of genomic binding has also been reported for other stress-inducible TFs (*e.g.*, JunB) [39], the decrease in ATF3 binding to a substantial number of genomic sites argues against the notion that the dynamic changes in ATF3 binding was a mere consequence of elevated ATF3 expression induced by DNA. As DNA damage can alter chromatin structure [43–45], it might allow access of some genomic sites to, while shielding other sites from, ATF3. Interestingly, the CPT treatment appeared to promote ATF3 to bind to sites distal to TSS (Fig. 5d). While the exact mechanism remains elusive, it might be that the epigenetic environments where the distal sites reside are favorable for TF binding. Indeed, these distal sites often coincide with p300/H3K27ac-enriched active enhancers (Fig. 3d), which are known to have lower nucleosomal density [5].

Like other TFs [36, 42], binding of ATF3 to the regulatory region of a gene did not always result in a change in gene expression. Indeed, although ATF3 bound more than 10,000 genes, a complete loss of ATF3 expression only altered expression of a small number of genes under both the quiescent and the stressed condition. While RNA-based assays (*e.g.*, microarray and RNA-seq) may not serve as accurate measurements of transcription activity [42], other TFs capable of binding the same motifs (*e.g.*, JunB) [39] might compensate for ATF3 loss. Interestingly, the ATF3-binding sites often contained motifs of other TFs in addition to the ATF3 motif (Fig. 2), suggesting that ATF3 might act in concert with other TFs to regulate gene expression. Our results also indicate that ATF3 can activate or repress gene expression depending on gene context. While the location and motif composition of the ATF3-binding site did not appear to determine whether ATF3 activates or represses gene expression (Additional file 1: Figure S1D and S1E), it is very likely that the epigenetic environment surrounding the ATF3-binding sites determine the availability of transcription co-activators (like Tip60), or transcription co-repressors (e.g., HDAC), which consequently transactivate or repress expression of ATF3-bound genes. Thus, the early notion that ATF3 homodimers and heterodimers respectively repress and activate transcription appears oversimplified and misleading.

An important finding from this study is that ATF3 bound to 37 % of genomic sites that were bound by p300 and characterized by high levels of H3K27ac under the basal condition (Fig. 3b). These genomic sites are defined as active enhancers and have been shown to contain functional regulatory elements that drive proximal gene expression during embryonic development [3, 4]. Interestingly, DNA damage increased the percentage of active enhancers bound by ATF3 to 57.6 %. Moreover, although ATF3 binding alone was not sufficient to regulate transcription, most of genes regulated by ATF3 appeared localized proximal to ATF3-bound active enhancers (Fig. 4). This strong correlation between TF binding to active enhancers and the regulation of gene expression was not without precedent. The transcription factor p63, for instance, was recently shown to bind H3K27ac-enriched active enhancers, and the binding correlates with dynamic gene expression regulated by p63 during epidermal differentiation [42]. As active enhancers often contain a cluster of motifs allowing for binding by multiple TFs, it is likely that these TFs collaboratively interact with the basal transcription machinery in core promoters to regulate gene expression. Therefore, the observed correlation between enhancer binding and transcriptional regulation is consistent with our notion that ATF3 needs to cooperate with other TFs to regulate gene expression.

The tumor suppressor p53 drives a transcription program for eliciting diverse cellular responses to DNA damage. Previously, we reported that ATF3 can activate p53 by binding and directly blocking its ubiquitination [16]. We also found that ATF3 can induce p53 activation by promoting the activity of a histone acetyltransferase Tip60 and the subsequent activation of ATM [22]. In this study, we revealed an additional mechanism by which ATF3 regulates p53, *i.e.*, co-localization with p53 at genomic sites. Indeed, we found that ATF3 was co-localized with p53 at more than 20 % of p53-binding sites identified by ChIP-seq (Fig. 6a). As ATF3 can interact with p53 [16], such co-localization might be a consequence of p53-mediated ATF3 recruiting (Fig. 6h), or vice versa (Fig. 6i). On the other hand, some co-localized genomic sites contained both the p53 and the ATF3 motif, and thus could be bound by p53 and ATF3 simultaneously. Regardless, close proximity between ATF3 and p53 at genomic sites might directly alter p53 conformation thereby regulating the p53 transcriptional activity (Fig. 6j). Our results are supported by a recent report, which carried out ATF3 ChIP-chip assays and shows binding of ATF3 to promoters of many known p53 target genes [46]. However, Our study indicates that a large number of co-localized sites were far beyond promoter regions [47] and were also often bound by p300. Therefore, the genomic co-localization of ATF3 and p53 serves as an additional mechanism for fine tuning p53 activity in the DNA damage response.

## Conclusions

Our results indicate that ATF3 likely preoccupies genomic sites regulatory for genes involved in the cellular stress response, and thus bookmarks these sites for transcriptional regulation under basal and stressed conditions.

## Methods

### Cell culture and generation of ATF3-knockout cells

HCT116 wild-type and p53-knockout cells (obtained from Bert Vogelstein) were cultured in McCoy’s 5A medium supplemented with 10 % fetal bovine serum. H1299 cells and 293 T cells were cultured in RPMI 1640 and DMEM medium, respectively. We knocked out ATF3 expression in HCT116 cells using a rAAV-based approach [23]. Briefly, left and right homology arms flanking a small region (22 bp) in the exon 3 of *ATF3* were amplified by PCR, and sequentially ligated into pAAV-TK-Acceptor [23] via restriction enzyme digestion. The resulted plasmid was then transfected into AAV-293 cells for rAAV packaging using the AAV Helper-free System (Agilent) following the manufacturer’s protocol. For viral infections, HCT116 cells in 60 mm dishes were incubated with 2 ml of viral supernatant overnight, followed by re-suspension in medium containing 500 μg/ml of G418 for selection. Genomic DNAs were then prepared from resistant single clones as describe previously [48], and used for PCR to identify targeted clones. To remove the inserted selection gene, targeted clones in 24-well plates were transfected with a Cre-expression plasmid. Single clones regaining G418 sensitivity were accordingly identified, and subjected to the 2nd round of genome editing to knock out the 2nd *ATF3* allele as describe above. The sequences of primers used in this report are available upon request.

### Chromatin immunoprecipitation

Chormatin immunoprecipitation was performed essentially as described previously [49]. Briefly, cells (2 × 10^7^) treated with or without 1.5 μM of CPT for 4 h were cross-linked with 1 mM of di(N-succinimidyl) glutarate (DSG) for 45 min, followed by 1 % formaldehyde for 10 min at room temperature. After treating with 0.125 M of glycine for 5 min, cells were resuspended in 10 ml of Solution I (10 mM Hepes-KOH, pH7.5, 10 mM EDTA, 0.5 mM EGTA, and 0.75 % Triton X-100), and incubated at 4 °C for 10 min. Cells were further incubated with 10 ml of Solution II (10 mM Hepes-KOH, pH7.5, 200 mM NaCl, 1 mM EDTA, and 0.5 mM EGTA) at 4 °C for 10 min before lysed in cold FA lysis buffer (50 mM Hepes-KOH, pH7.5, 140 mM NaCl, 1 mM EDTA, 1 % Triton X-100, 0.1 % sodium deoxycholate, and proteinase inhibitors). Chromatin was sheared by sonication using Bioruptor to an average fragment size of 500 bp, and then incubated with 2 μg of the antibody (ATF3, sc-188; p53, sc-126; p300, sc-585) or normal IgG (rabbit, sc-3888; mouse, sc-2025) purchased from Santa Cruz, at 4 °C overnight. Immunocomplexes were precipitated with 30 μl of ssDNA-protein A/G agarose (Millipore) at 4 °C for 2 h, and sequentially washed with Buffer I (50 mM Tris–HCl, pH8.0, 150 mM NaCl, 1 % SDS, 0.5 % sodium deoxycholate, 1 % NP 40, and 1 mM EDTA), Buffer II (buffer I with 500 mM NaCl), Buffer III (50 mM Tris–HCl, pH8.0, 250 mM LiCl, 0.5 % sodium deoxycholate, 1 % NP 40, and 1 mM EDTA), and TE buffer (50 mM Tris–HCl, pH8.0, and 1 mM EDTA). Bound chromatin was eluted with 0.3 ml of Elution buffer (50 mM Tris–HCl, pH8.0, 1 % SDS, and 1 mM EDTA). After reversal of crosslinking, RNase A and Proteinase K was added, and DNA was purified by phenol extraction and ethanol precipitation. For re-ChIP assays, chromatin immunoprecipitated with the ATF3 antibody was eluted in 0.15 ml of Elution buffer, and then diluted by 20 times with re-ChIP buffer (20 mM Tris–HCl, pH8.0, 150 mM NaCl, 1 % Triton X-100, and 2 mM EDTA), followed by incubation with the p53 antibody as described above.

### ChIP-seq and data analysis

ChIP-seq libraries were prepared according to standard protocols using Biosicentific’s DNA Sample Kit (cat#514101) [50]. Libraries were sequenced using Illumina Hi-Seq platforms. Sequence reads were aligned to the Human Reference Genome (assembly hg19) using Burrows-Wheeler Alignment (BWA) Tool Version 0.6.1. Peak identification, overlapping, subtraction and feature annotation of enriched regions were performed using Hypergeometric Optimization of Motif EnRichment suite (HOMER). Heatmaps and intensity plots of peaks were generated by Perl script, R and/or java Treeview. HOMER was used to check motif enrichment.

### Microarray data analysis

Total RNA was prepared using Agilent Total RNA Isolation Mini Kit (cat# 5185–6000). Microarray expression profiling was performed using HumanHT-12 v 4.0 Expression BeadChip (Illumina). Data were preprocessed and normalized by GenomeStudio. Differentially expressed genes were identified by Bioconductor limma package and GenePattern. Heatmap view of differentially expressed genes was created by Cluster and Java Treeview. GO term enrichment was determined using DAVID.

### Western blotting and quantitative PCR

Western blotting assays were performed as described previously [16]. In brief, cells were lysed in RIPA buffer containing 50 mM Tris–HCl, pH 7.4, 1 % Nonidet P-40, 0.25 % sodium deoxycholate, 150 mM NaCl, 1 mM EDTA, 1 mM PMSF, and 1 mM NaF, 1 mM Na_3_VO_4,_ and protease inhibitor cocktail (Roche), and then resolved in SDS-polyacrylamide electrophoresis for immunoblotting. Quantitative PCR assays were carried out using SYBR Green as described elsewhere [49].

### Availability of supporting data

The data sets supporting the results of this article are available in the GEO with the accession number GSE74363 (http://www.ncbi.nlm.nih.gov/geo/query/acc.cgi?token=yfozycagbfojfkz&acc=GSE74363).

## Additional files

Additional file 1: This pdf file contains Figure S1, S2 and S3. (PDF 531 kb) Additional file 2: Table S1. A list of unique genes containing overlapped ATF3 and p53 peaks where both ATF3 and p53 motifs are present. (XLSX 32 kb)
