# Supplementary material for: The common stress responsive transcription factor ATF3 binds genomic sites enriched with p300 and H3K27ac for transcriptional regulation
Source: BMC Genomics. 2016 May 4;17:335. doi: 10.1186/s12864-016-2664-8 (PMC4857411; doi:10.1186/s12864-016-2664-8)
Supplement: Additional file 1: — This pdf file contains Figure S1, S2 and S3. (PDF 531 kb) [file 12864_2016_2664_MOESM1_ESM.pdf]

Supplemental Figures

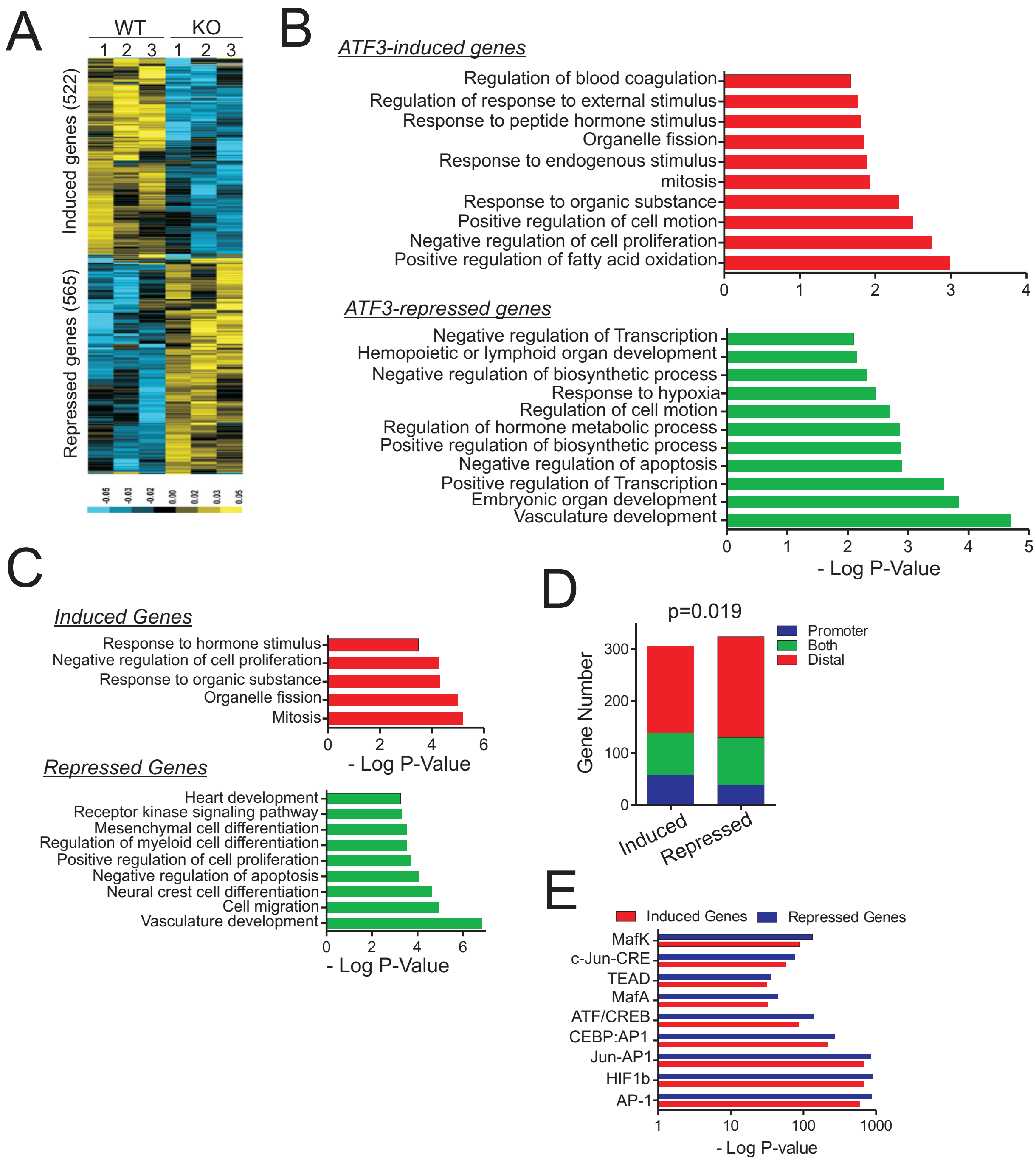

Figure S1. **Analyses of genes differentially expressed between ATF3-wildtype and knockout cells.** (**A**) Heatmap showing differentially-expressed genes derived from microarray data. (**B**) GO analysis of ATF3-induced, and repressed genes. (**C**) GO analysis of genes directly regulated by ATF3. (**D**) The binding of ATF3 to distal regions tended to repress gene expression. The p value was calculated by the Fisher Exact test. (**E**) No difference was found in the motif composition of ATF3-binding sites between ATF3-induced and ATF3-repressed genes.

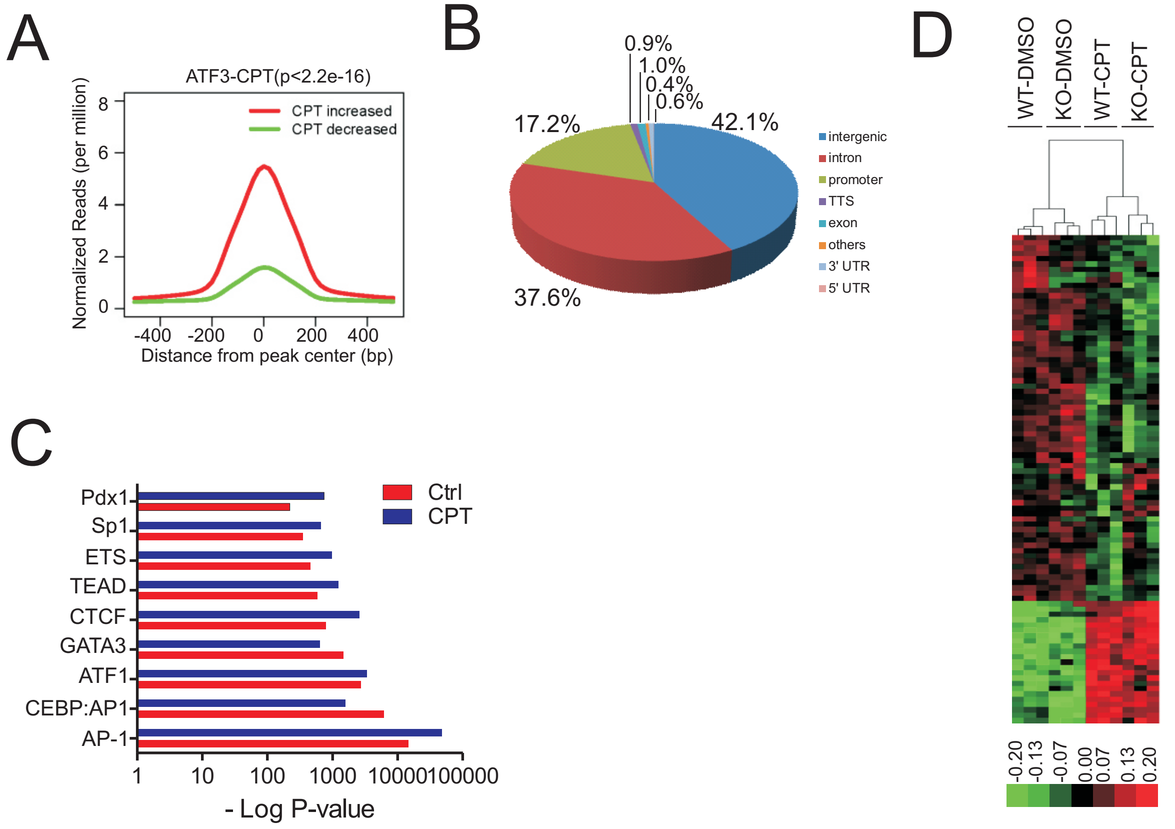

Figure S2. **Profiling ATF3-binding sites under the stressed condition.** (**A**) Intensity plot showing ATF3 peaks increased or decreased by CPT under the stressed condition. (**B**) Distribution of ATF3-binding sites under the stressed condition. (**C**) Comparison of motifs contained in ATF3-binding sites between the quiescent (Ctrl) and stressed (CPT) condition. (**D**) Heatmap of genes regulated by ATF3 under the DNA damage condition.

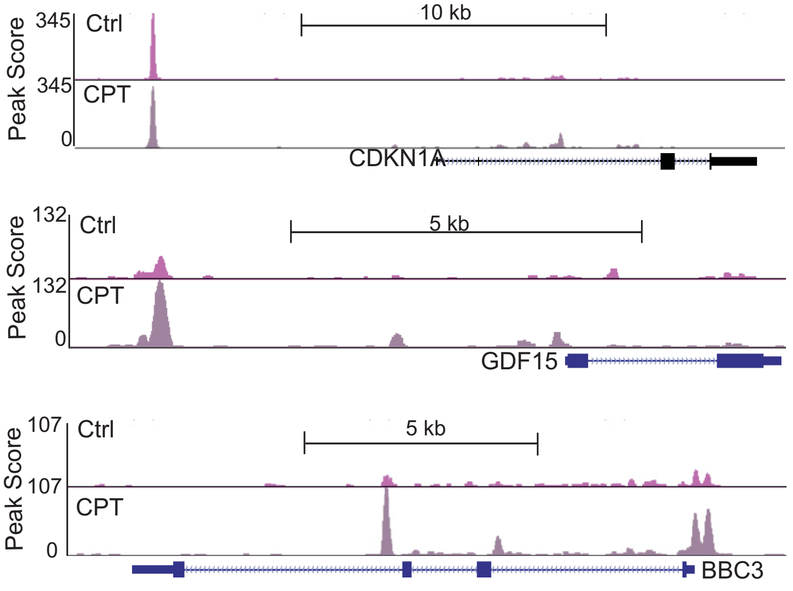

Figure S3. **ATF3 binding before and after CPT treatments.**
